# Insurance Delays in Initiation of Tumor Necrosis Factor Inhibitors in Children With Juvenile Idiopathic Arthritis

**DOI:** 10.1001/jamanetworkopen.2022.8330

**Published:** 2022-04-21

**Authors:** Jordan E. Roberts, Mary Fan, Mary Beth F. Son

**Affiliations:** 1 Boston Children’s Hospital, Harvard Medical School, Boston, Massachusetts

## Abstract

This cohort study compares the delays in tumor necrosis factor inhibitor (TNFi) initiation because of insurance among children enrolled in public and private insurance plans.

## Introduction

Prompt escalation to tumor necrosis factor inhibitors (TNFis) is recommended for juvenile idiopathic arthritis (JIA) refractory to disease-modifying antirheumatic drugs (DMARDs).^1^ Increasingly, TNFis are used as first-line therapy, and early treatment may be associated with better outcomes.^2^ Although insurance is associated with treatment delays in adults with rheumatoid arthritis,^3,4^ it is unknown if prior authorization (PA) requirements delay TNFi initiation among children with JIA. We aimed to characterize delays in TNFi initiation because of insurance among children enrolled in public plans vs private plans.

## Methods

This cohort study followed the Strengthening the Reporting of Observational Studies in Epidemiology (STROBE) reporting guideline and was determined to be exempt from review by the institutional review board at Boston Children's Hospital. Informed consent was waived because the study was determined to have minimal risk to the privacy of the patients.

We compared TNFi initiation between children (ie, younger than 21 years) who were publicly and privately insured with new diagnoses of JIA from 2018 to 2019 at Boston Children’s Hospital. Patient demographics and insurance plan information were obtained via automated extraction from the electronic medical record. We compared public and private insurance outcomes using Wilcoxon tests, *t* tests, and Fisher exact tests. The Wilcoxon tests and *t* tests were 2-sided, and statistical significance was set at *P* < .05. All analyses were conducted using R statistical software version 4.0.3 (R Project for Statistical Computing) and were completed between November 2021 and February 2022. Additional details about our methods are shown in the eMethods in the Supplement.

## Results

We identified 54 children and young adults prescribed TNFi for JIA; these patients had a mean (SD) age of 9.9 (5.9) years, 31 (57%) were female, and 44 (81%) had private insurance (Table 1). At the time of the TNFi PA request, 49 children (91%) had received DMARDs, and 31 (61%) had received nonsteroidal anti-inflammatory drugs (NSAIDs). PA for TNFi was required for 53 (98%) plans; 14 PA requests (26%) were denied and required written appeal or peer-to-peer review. Median (IQR) time to approval was 3 (1-11.25) days in publicly and privately insured, though 12 (22%) had delays of 2 weeks or longer. TNFi initiation took more than 30 days for more than 25% of the requests (Table 2). Among those with PA denials requiring written appeal or peer-to-peer, median (IQR) approval time was 15.5 days (11.2-24.5) vs 4.8 (1-5.8) days in those whose initial PAs were approved without written appeal or peer-to-peer (*P* < .001). The median (IQR) time to TNFi initiation was 34.5 (29.8-46.2) days among those with PA denials requiring written appeal or peer-to-peer vs 16.5 (10.2-27.2) days (*P* < .02).

**Table 1.** Demographic and Disease-Related Characteristics by Patients’ Insurance Status

Characteristic	Patients, No. (%)			*P* value
	All (N = 54)^a^	Public (n = 10)	Private (n = 44)	
Age, mean (SD), y	9.9 (5.9)	10.5 (6.2)	9.9 (5.9)	.77
Sex				
Female	31 (57)	3 (30)	28 (64)	.08
Male	23 (43)	7 (70)	16 (36)	.08
Race				
Asian and White	1 (2)	0	1 (2)	<.001
Black	5 (9)	2 (20)	3 (7)	
White	34 (63)	2 (20)	32 (73)	
Declined to answer	7 (13)	0	7 (16)	
Other^b^	6 (11)	6 (60)	0	
Unable to answer	1 (2)	0	1 (2)	
Hispanic	4 (7)	3 (30)	1 (2)	.02
Non-English primary language	4 (7)	4 (40)	0	<.001
Patient portal access enabled	38 (70)	6 (60)	32 (73)	.46
Arthritis subtype (n = 39)				
Oligoarticular				
Persistent	13 (28)	1 (12.5)	12 (31)	.23
Extended	1 (2)	0	1 (3)	
Polyarticular				
RF positive	7 (15)	1 (12.5)	6 (15)	
RF negative	7 (15)	2 (25)	5 (11)	
Psoriatic arthritis	12 (26)	1 (12.5)	11 (28)	
Enthesitis related arthritis	6 (12)	2 (25)	4 (10)	
Undifferentiated arthritis	1 (2)	1 (12.5)	0	
Active joint count, median (IQR) (n = 47)	2 (1-4)	4 (2.75-4)	2 (1-3)	.10
Parent/patient global assessment, median (IQR) (n = 35)^c^	3 (2.5-5)	3 (3-3)	3.5 (2.25-5)	.39
Physician global assessment, median (IQR) (n = 46)^c^	3 (1.25-4)	3.5 (2.75-4)	3 (1-4)	.46
Prior or current NSAID use at time of PA	33 (61)	8 (80)	25 (57)	.28
Prior or current DMARD use at time of PA	49 (91)	9 (90)	40 (91)	>.99

Abbreviations: DMARD, disease-modifying antirheumatic drug; NSAID, nonsteroidal anti-inflammatory drugs; PA, prior authorization; RF, rheumatoid factor.

^a^ Three patients with both public and private insurance are included in the private insurance group.

^b^ Other race and ethnicity includes all patients who self-reported this as their race or ethnicity category at time of hospital clinic registration.

^c^ Visual analogue scale from 1 to 10 with 10 denoting greater impact of disease.

**Table 2.** Prior Authorization and Approval Timeline by Insurance Status

Timeline	Patients, No. (%)		
	All (N = 54)^a^^,^^b^	Public insurance (n = 10)	Private insurance (n = 44)
Prior authorization time to approval, median (IQR), d	3 (1-11.25)	2.5 (1-18.8)	3 (1-9.75)
Time from recommendation to start tumor necrosis factor inhibitor to first dose, median (IQR), d	24 (13-34)	20 (11-44)	24 (13-33)
Initial prior authorization request denied	14 (26)	3 (30)	11 (25)
Written appeal required	12 (22)	3 (30)	9 (21)
Peer-to-peer required	2 (4)	1 (10)	1 (2)
Started different medication because of denial	4 (7)	0	4 (9)

^a^ Three patients with both public and private insurance are included in the private insurance group.

^b^ *P* > .05 for all.

Among publicly insured individuals, reasons for PA denial included step therapy with DMARD, lack of medical necessity, and additional information needed. Among privately insured individuals, reasons included step therapy with NSAID, submission to different insurance for a dually insured patient, non-FDA approved indication, patient age, requirement for different TNFi, and additional information. Among the 4 children whose denials were not overturned, all were privately insured and were required to use adalimumab instead of etanercept or infliximab as their rheumatologist recommended.

## Discussion

Children with public and private insurance commonly experienced delays in TNFi initiation because of PA requirements and denials. Although denials were common, all were eventually approved for a TNFi, suggesting that utilization management strategies present barriers to care despite appropriate specialty medication requests, which is consistent with work in adults.^5^

Limitations of this study include the use of a single center, which may not be generalizable to insurance plans throughout the US. Comparable delays between plan types may reflect relatively comprehensive Medicaid coverage in Massachusetts or formulary restrictions and underinsurance among privately insured individuals.^6^

Our findings highlight barriers to specialty medications for children. Currently, no TNFi is FDA-approved for JIA in children under 2, although onset in toddlers is common. Requirements to use specific TNFis may differentially impact children. Uveitis, a pediatric JIA complication, is effectively treated with adalimumab or infliximab, but not etanercept. Infliximab’s intravenous formulation allows for more precise weight-based dosing than subcutaneously administered TNFis. Delays in insurance approval were a common barrier to TNFi initiation in our cohort. Further research about the impact of treatment delays on JIA outcomes in larger cohorts is needed.

## Notes

Supplement.
eMethods.
