# Supplementary material for: Insurance Delays in Initiation of Tumor Necrosis Factor Inhibitors in Children With Juvenile Idiopathic Arthritis
Source: JAMA Netw Open. 2022 Apr 21;5(4):e228330. doi: 10.1001/jamanetworkopen.2022.8330 (PMC9024383; doi:10.1001/jamanetworkopen.2022.8330)
Supplement: Supplement. — eMethods. [file jamanetwopen-e228330-s001.pdf]

Supplemental Online Content

Roberts JE, Fan M, Son MBF. Insurance delays in initiation of tumor necrosis factor inhibitors in children with juvenile idiopathic arthritis. *JAMA Netw Open*. 2022;5(4):e228330. doi:10.1001/jamanetworkopen.2022.8330

eMethods.

This supplemental material has been provided by the authors to give readers additional information about their work.

© 2022 Roberts JE et al. *JAMA Network Open.*

eMethods.

Case Identification and Data Extraction

Patients with potential JIA diagnosis were identified via electronic medical record search using the following ICD-10 codes: M05, M05.6, M05.7, M05.8, M06.0, M06.4, M06.9, M08, M45, L40.54, and M13.

Once identified via ICD-10 code, dates of first prescription or infusion of infliximab, adalimumab, and etanercept were automatically extracted. Charts were manually reviewed to confirm JIA diagnosis based on rheumatologist's clinic note, initiation or non-initiation of TNFi inhibitor, and date of TNFi initiation. Those with systemic-onset JIA were excluded. Dates of prior approval (PA) requests and denials, requirements for appeals or peer-to-peers, and reasons for PA denial were extracted from medical notes. Initial review of charts was conducted by MF, and the information extracted was confirmed by JER. Patient demographics and insurance plan information were obtained via automated extraction from the electronic medical record. Rheumatology clinic notes of all children with an ICD-10 code for JIA from 2018-2019 but without chart-confirmed TNFi initiation were searched using HoundDog software for the terms "adalimumab," "Humira," "infliximab," "Inflectra," "Remicade," "etanercept," and "Enbrel." All notes identified in the search were then manually reviewed by MF and JER to identify children for whom a TNFi was recommended by their rheumatologist, but not initiated. Reasons for non-initiation were recorded.

© 2022 Roberts JE et al. *JAMA Network Open.*
